# Constant Light Desynchronizes Olfactory versus Object and Visuospatial Recognition Memory Performance

**DOI:** 10.1523/JNEUROSCI.3213-16.2017

**Published:** 2017-03-29

**Authors:** Shu K.E. Tam, Sibah Hasan, Harry M.C. Choi, Laurence A. Brown, Aarti Jagannath, Steven Hughes, Mark W. Hankins, Russell G. Foster, Vladyslav V. Vyazovskiy, David M. Bannerman, Stuart N. Peirson

**Affiliations:** ^1^Sleep and Circadian Neuroscience Institute (SCNi), Nuffield Department of Clinical Neurosciences, Oxford Molecular Pathology Institute, Sir William Dunn School of Pathology, University of Oxford, Oxford OX1 3RE, United Kingdom,; ^2^Department of Experimental Psychology, University of Oxford, Oxford OX1 3PH, United Kingdom, and; ^3^Department of Physiology, Anatomy and Genetics, University of Oxford, Oxford OX1 3QX, United Kingdom

**Keywords:** circadian, clock genes, hippocampus, internal desynchrony, olfactory bulb, suprachiasmatic nuclei

## Abstract

Circadian rhythms optimize physiology and behavior to the varying demands of the 24 h day. The master circadian clock is located in the suprachiasmatic nuclei (SCN) of the hypothalamus and it regulates circadian oscillators in tissues throughout the body to prevent internal desynchrony. Here, we demonstrate for the first time that, under standard 12 h:12 h light/dark (LD) cycles, object, visuospatial, and olfactory recognition performance in C57BL/6J mice is consistently better at midday relative to midnight. However, under repeated exposure to constant light (*r*LL), recognition performance becomes desynchronized, with object and visuospatial performance better at subjective midday and olfactory performance better at subjective midnight. This desynchrony in behavioral performance is mirrored by changes in expression of the canonical clock genes *Period1* and *Period2* (*Per1* and *Per2*), as well as the immediate-early gene *Fos* in the SCN, dorsal hippocampus, and olfactory bulb. Under *r*LL, rhythmic *Per1* and *Fos* expression is attenuated in the SCN. In contrast, hippocampal gene expression remains rhythmic, mirroring object and visuospatial performance. Strikingly, *Per1* and *Fos* expression in the olfactory bulb is reversed, mirroring the inverted olfactory performance. Temporal desynchrony among these regions does not result in arrhythmicity because core body temperature and exploratory activity rhythms persist under *r*LL. Our data provide the first demonstration that abnormal lighting conditions can give rise to temporal desynchrony between autonomous circadian oscillators in different regions, with different consequences for performance across different sensory domains. Such a dispersed network of dissociable circadian oscillators may provide greater flexibility when faced with conflicting environmental signals.

**SIGNIFICANCE STATEMENT** A master circadian clock in the suprachiasmatic nuclei (SCN) of the hypothalamus regulates physiology and behavior across the 24 h day by synchronizing peripheral clocks throughout the brain and body. Without the SCN, these peripheral clocks rapidly become desynchronized. Here, we provide a unique demonstration that, under lighting conditions in which the central clock in the SCN is dampened, peripheral oscillators in the hippocampus and olfactory bulb become desynchronized, along with the behavioral processes mediated by these clocks. Multiple clocks that adopt different phase relationships may enable processes occurring in different brain regions to be optimized to specific phases of the 24 h day. Moreover, such a dispersed network of dissociable circadian clocks may provide greater flexibility when faced with conflicting environmental signals (e.g., seasonal changes in photoperiod).

## Introduction

Virtually all organisms possess an internal circadian clock that adapts physiology and behavior to the varying demands of the 24 h day. In mammals, the suprachiasmatic nuclei (SCN) of the anterior hypothalamus are the site of the master circadian pacemaker and lesions of the SCN abolish daily rhythms in behavior and endocrine function (Moore and Eichler, 1972; Stephan and Zucker, 1972). The SCN clock is based upon an intracellular transcriptional–translational feedback loop involving a number of canonical clock genes (Reppert and Weaver, 2002). This includes *Period1* (*Per1*) and *Period2* (*Per2*), which are directly modulated by light to enable the SCN clock to be entrained to the external light/dark (LD) cycle (Albrecht et al., 1997; Bae et al., 2001). Rhythmic clock gene expression is also found in many different regions of the brain outside of the SCN (Guilding and Piggins, 2007; Herzog, 2007; Kyriacou and Hastings, 2010). These peripheral clocks are thought to regulate local rhythms in tissue-specific physiology, but are dependent upon the master pacemaker in the SCN to prevent internal desynchrony and maintain an appropriate phase relationship with the external LD cycle (Herzog and Tosini, 2001; Dibner et al., 2010). In isolation from the SCN, most peripheral clocks rapidly dampen as individual cellular oscillators become desynchronized (Yoo et al., 2004).

Like many biological and cognitive processes, learning and memory are under the influence of the circadian system and circadian variation in performance has been described in many nonmammalian species, with performance better during the active phase (Fernandez et al., 2003; Lyons et al., 2005). In contrast, findings from nocturnal rodents are less conclusive. Some studies reported better performance during the active (i.e., dark) phase (Valentinuzzi et al., 2001; Gritton et al., 2009, 2012), whereas others found better performance during the light phase (Chaudhury and Colwell, 2002; Rawashdeh et al., 2014). These contradictory findings could be due to the nature of the behavioral tasks used (typically appetitive or aversive) or the nature of stimuli used and have been a major limitation in further understanding the mechanisms by which circadian rhythms regulate learning and memory performance.

Here, we use the spontaneous object recognition task (Ennaceur and Delacour, 1988; Dix and Aggleton, 1999; Bevins and Besheer, 2006) to investigate circadian variation in memory performance in C57BL/6J mice. Different variants of the task are used to investigate memory for objects, places, and odors to determine whether there is any consistent day/night variation in memory for different types of stimulus. We show that, under standard 12 h:12 h LD cycles, memory performance for objects, places, and odors is consistently better during the light phase. We then repeated these experiments under constant conditions, using 48 h of constant light (LL) interspersed with 48 h of LD to prevent free running. Under these repeated LL (*r*LL) conditions, in which environmental time cues are eliminated at the time of recognition testing, we demonstrate a decoupling of object and visuospatial performance (which remains optimal during the subjective light phase) versus olfactory performance (which now becomes optimal during the subjective dark phase). Moreover, we show that circadian changes in mRNA levels of *Per1* and the immediate-early gene *Fos* are dampened in the SCN. Strikingly, in accordance with the behavioral data, *r*LL alters the circadian phase of *Per1* and *Fos* expression in the olfactory bulb, but not in the dorsal hippocampus, a region that (together with adjacent and interconnected cortical areas) contributes to object and visuospatial recognition memory processes (Clark et al., 2000; Broadbent et al., 2010; Brown et al., 2010; Aggleton et al., 2012; Cohen et al., 2013). Collectively, our data provide a unique demonstration that, under abnormal lighting conditions in which SCN rhythms are dampened, decoupling of olfactory versus object and visuospatial recognition performance can occur. These dissociable circadian rhythms in behavioral performance may provide greater flexibility when faced with conflicting environmental signals (e.g., seasonal changes in photoperiod).

## Materials and Methods

### Animals

C57BL/6JOlaHsd male mice (2.5–5 months of age; Envigo RRID:IMSR_JAX:000664) were used in all experiments. Upon arrival at the colony, mice were put in pairs in plastic cages (length × width × height = 46 cm × 29 cm × 23 cm) with *ad libitum* access to food and water and housed under LD; the room temperature of the colony was maintained at 22 ± 2°C. Cages were placed inside a light-tight ventilated chamber (LTC) to eliminate exposure to extraneous photic cues (Fisher et al., 2012). The LTC was equipped with multiple white LEDs, which provided a light level of 250–280 lux (measured at the center of the cage floor) in the light phase. All mice were housed under LD for at least 7 d before they were allocated to different lighting conditions (described below). All procedures were performed in accordance with the United Kingdom Animals (Scientific Procedures) Act 1986 under Project Licenses 30/2812 and 30/3371 and Personal License I869292DB.

### Lighting conditions

#### Condition 1: standard 12 h:12 h LD cycles

**Figure 1.**
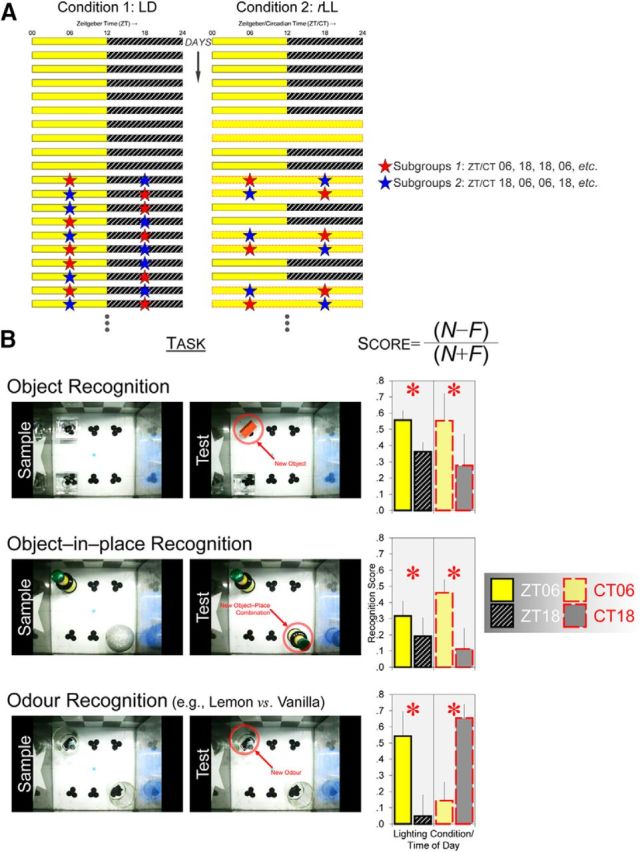
*r*LL desynchronizes object and visuospatial versus odor recognition memory performance. ***A***, Under the standard 12 h:12 h LD condition (left), each mouse was given different types of recognition task repeatedly at midday (ZT 6) and midnight (ZT 18). Under *r*LL (right), the lighting condition was alternated between 2 d of LD and 2 d of LL and mice were tested at subjective midday (CT 6) and at subjective midnight (CT 18) starting from the second cycle of LL. Red and blue stars show the different time points at which recognition tasks were given. Mice in Subgroup 1 were given different recognition tasks at ZT/CT 6, 18, 18, 6, 18, 6, 6, 18, etc., across days, whereas Subgroup 2 received the reverse arrangement (i.e., ZT/CT 18, 6, 6, 18, etc.). ***B***, Under LD, performance in the object, object-in-place, and odor recognition tasks was consistently better at ZT 6 than at ZT 18. Under *r*LL, object and visuospatial performance was better at CT 6 than at CT 18, similar to the results under LD (top and middle rows); asterisks (*p < 0.05) indicate main effects of time of day on recognition scores (*N* − *F*)/(*N* + *F*), where *N* and *F* represent novel versus familiar objects, object–place combinations, or odors. However, odor recognition performance was phase shifted and became better at CT 18 than at CT 6, resulting in a significant lighting condition × time of day interactive effect on odor recognition scores (bottom row); asterisks (*p < 0.05) indicate simple main effects of time of day. In the object recognition task, *n* = 32 under LD and *n* = 8 under *r*LL; in the object-in-place task, *n* = 16 under LD and *n* = 16 under *r*LL; in the odor recognition task, *n* = 8 under LD and *n* = 24 under *r*LL. Error bars indicate SEM.

For mice in the LD condition, behavioral testing was conducted at midday [Zeitgeber time (ZT) 6 h ± 30 min] and at midnight (ZT 18 h ± 30 min) across multiple days, as shown in Figure 1*A*, left. ZT 0 h indicates the onset of the light phase and ZT 12 h indicates the onset of the dark phase.

#### Condition 2: *r*LL

For mice in the *r*LL condition, the lighting condition was alternated between standard LD and 48 h of constant light, as shown in Figure 1*A*, right. Behavioral testing was conducted at the animal's subjective midday [Circadian time (CT) 6 h ± 30 min] and subjective midnight (CT 18 h ± 30 min) under multiple 48 h periods of constant light (starting from the second cycle of LL); the circadian time points are defined by the LD transition before the onset of LL. The repeated alternation between standard LD and LL enables animals to reentrain to the original LD cycle after 2 d of LL (Thompson et al., 2008), preventing animals from adopting different phases at the time of testing due to free running under prolonged LL (which will result in an internal period of longer than 24 h; Aschoff, 1960). Moreover, LL was used rather than constant dark (DD) because visuospatial recognition requires visual input (Tam et al., 2016) and could not be assessed in DD.

#### Control LL

**Figure 2.**
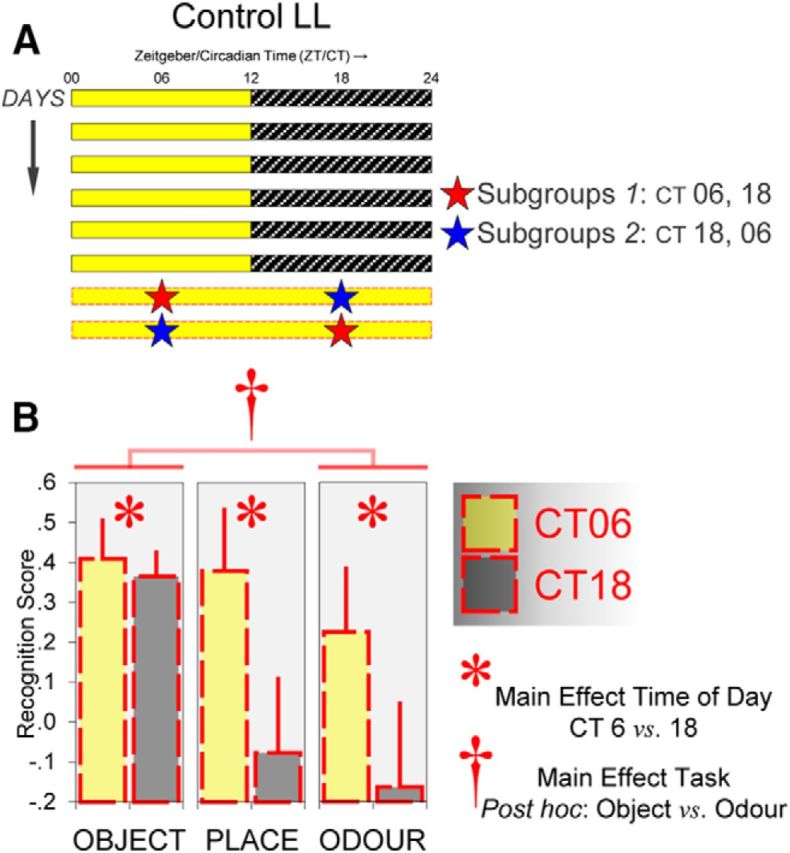
A single 48 h period of light (control LL) is not sufficient to desynchronize object and visuospatial versus odor recognition memory performance. ***A***, Under control LL, each mouse was given a one-off 48 h period of light and received the object, object-in-place, or odor recognition task at CT 6 and 18 in a counterbalanced order. Mice in Subgroup 1 were given the recognition task first at CT 6 and subsequently at CT 18, whereas in Subgroup 2 mice were tested first at CT 18 and subsequently at CT 6. ***B***, Performance in the object, object-in-place, and odor recognition tasks was consistently better at CT 6, as indicated by the main effect of time of day (*p < 0.05). The task × time of day interaction was not significant, indicating that control LL did not induce a significant phase shift in odor recognition performance. However, performance in the odor task was generally poorer than performance in the object task (†Bonferroni *post hoc* comparison, *p* < 0.05). In the object and odor recognition tasks, *n* = 16 per task; in the object-in-place task, *n* = 8. Error bars indicate SEM.

A one-off period of LL was used as a control condition to investigate whether the effects observed under *r*LL were due to repeated alternation between LD and LL or just the prolonged exposure to light. Mice in this condition were exposed to a single 48 h period of LL, as shown in Figure 2*A* and behavioral testing was conducted at their subjective midday (CT 6 h ± 30 min) and subjective midnight (CT 18 h ± 30 min), defined by the LD transition before the onset of LL.

### Apparatus and stimuli in recognition tasks

#### Arena

A 20 cm × 20 cm × 20 cm open-top arena made of transparent acrylic was used for recognition testing. To facilitate discrimination of the four corners, two distinct 20 cm × 20 cm wallpapers were attached to the outside of the arena (Fig. 1*B*). One of the wallpapers was a checkerboard pattern with alternating 4 cm × 4 cm black and white squares. Each square subtended a visual angle of 23° vertically and horizontally (assuming the mouse's eye at the center of the arena). The other wallpaper had a white symmetrical five-point star on a black background. The star shape was drawn within a notational circle with a diameter of 14 cm, which subtended a visual angle of 70°. Small pieces of hook-and-loop stickers (Rip 'n' Grip) were adhered to the floor of the arena and to the bottom of all objects so that objects could be affixed to the arena during behavioral testing. A web camera was positioned 37 cm above the center of the arena. The arena was placed inside a LTC equipped with white LEDs, which provided a light level of 120–150 lux at the center of the arena for all recognition tasks.

#### Objects

For object and visuospatial recognition trials, we used everyday objects (e.g., bottles, light bulbs, and paperweights; Fig. 1*B*, top row) that had a base area smaller than 7.0 cm × 7.0 cm and a height <9 cm. The objects to be discriminated on each object recognition trial differed in multiple sensory dimensions such as color, size, shape, and texture so they could be discriminated on the basis of visual cues, nonvisual cues, or both. A detailed description of some of the objects used in the current study can be found in Tam et al. (2016). There were at least four replicates of each object so that different replicates of the same object could be presented in sample and test phases, eliminating the possibility that, at test, the mouse simply recognized and ignored its own odor traces left on the object at preexposure.

#### Odors

For odor recognition trials, various essential oils, including lemon, vanilla, and peppermint extracts (Dr. Oetker), orange, chocolate, and rose extracts (Nielsen-Massey), as well as banana and jasmine essence (Double Seahorse), were used as olfactory stimuli. Shortly before recognition testing, 1 ml of each stimulus was delivered with a syringe into a clear shot glass (Fig. 1*B*, bottom row) with a base diameter of 4.6 cm and a height of 6.4 cm. There were eight identical shot glasses so that different shot glasses could be presented in the sample and test phases.

### Behavioral procedure

Before recognition testing, all mice were kept under LD for at least 7 d and given 6 habituation trials in the empty arena over a 3 d period: 3 trials at ZT 6 and 3 trials at ZT 18. The aim of these trials was to minimize the effects of arousal associated with handling and novelty of the arena, which could otherwise interfere with object exploration and disrupt test performance. At ZT 6 and 18, the mouse was removed from its home cage and put into the arena. The time required to transfer the mouse from its home cage into the arena was 10–20 s only because the apparatus was set up in a separate LTC located right below the LTC where animals were housed; therefore, mice received relatively little handling before each trial. Mice were allowed to explore the empty arena for 10 min on each habituation trial. After the habituation phase, each mouse in LD or *r*LL was tested repeatedly in different variants of the spontaneous recognition task across diurnal or circadian cycles in different sequences (Table 1). In the control condition, in which there was only a single 48 h period of LL, each mouse was given the object, visuospatial, or odor task at CT 6 and 18.

**Table 1.** Task sequence in each cohort

Lighting condition	Cohort	Recognition task sequence
Condition 1: LD	1A (*n* = 8)	H × 6 → (1) object × 4 → (2) object displacement × 2 → (3) object-in-place × 2
	1B (*n* = 8)	H × 6 → (1) object × 4 → (2) object displacement × 2 → (3) object-in-place × 2 → (4) odor × 4
	1C*^a^* (*n* = 8)	H × 6 → (1) object × 4 → (2) object displacement × 2 → (3) object-in-place × 2
Condition 2: *r*LL	2A*^b^* (*n* = 8)	H × 6 → (1) object displacement × 2 → (2) object-in-place × 2 → (3) odor × 4
	2B*^c^* (*n* = 8)	H × 6 → (1) odor × 4 → (2) object displacement × 2 → (3) object-in-place × 2 → (4) object × 4
	2C*^b^* (*n* = 8)	H × 6 → (1) object displacement × 2 → (2) odor × 2
Control LL	3A (*n* = 16)	H × 6 → (1) object × 2
	3B (*n* = 8)	H × 6 → (1) object-in-place × 2
	3C (*n* = 16)	H × 6 → (1) object × 2 (LD) → (2) odor × 2 (LL)

All mice received 6 habituation trials (H) in the empty arena (3 trials at ZT 6 and 3 trials at ZT 18 under LD) before recognition testing. In each cohort, half of the recognition trials were given at ZT/CT 6 and the remaining trials were given at ZT/CT 18.

*^a^*Sample–test delay was 24 h for cohort 1C; for all other cohorts, the sample–test delay was 5 min.

*^b^*Cohorts 2A and 2C experienced one cycle of LD–LL alternation before recognition testing.

*^c^*Cohort 2B experienced four cycles of LD–LL alternation before recognition testing.

#### Object recognition

The object recognition task consisted of a sample phase and a test phase separated in time by either a 5 min or 24 h delay for mice under LD. For mice under *r*LL and control LL, the sample–test delay was always 5 min. At ZT/CT 6 or 18, a mouse was removed from its home cage and put into the arena. Two identical replicates of an object, *A*_1_ and *A*_2_, were placed at two corners of the arena (top left and bottom left) and the animal was allowed to explore the objects and arena freely for 10 min. After the required delay, the animal was tested for 1 min in the same arena (which was cleaned with 50% ethanol). One replicate of *A* was replaced by a novel object, *B*, whereas the other replicate of *A* was replaced by a third replicate, *A*_3_, which had never been presented to the animal. The time of day at which the object recognition task was given was counterbalanced under each lighting condition. For example, half of the mice under LD were given the object recognition task at ZT 6, ZT 18, ZT 18, and ZT 6 (Subgroup 1, red stars in Fig. 1*A*, left), whereas for the remaining mice, the task was given at ZT 18, ZT 6, ZT 6, and ZT 18 (Subgroup 2, blue stars in Fig. 1*A*, left). The same counterbalancing procedure was applied in *r*LL and control LL. A different pair of objects was used on each of the four object recognition trials.

As each mouse was tested at both (subjective) midday and midnight, the identities of the novel and familiar objects were counterbalanced across the two different time points. For example, for animals given the object recognition task first at ZT 6 and then at ZT 18 (Subgroup 1, red stars in Fig. 1*A*, left), a wood block and a glass candle holder were used at ZT 6 and a plastic bottle and a spherical ornament were used at ZT 18. This arrangement was reversed for animals given the object recognition task first at ZT 18 and then at ZT 6 (Subgroup 2, blue stars in Fig. 1*A*, left), the wood block and glass candle holder at ZT 18 and the plastic bottle and spherical ornament at ZT 6. In addition, within each subgroup at a particular time of day, the identities of novel and familiar objects and their positions at test were counterbalanced to take into account any potential bias toward a particular object or a certain part of the arena. For example, for Subgroup 1, which were first tested at ZT 6 (or Subgroup 2, which were first tested at ZT 18), the wood block was assigned as the familiar stimulus at test and the glass candle holder as the novel stimulus for half of the mice; this arrangement was reversed for the remaining half of animals such that the candle holder was the familiar object and the wood block was the novel object at test. Furthermore, for half of the animals within each of these subgroups, the novel object was located at the top left corner of the arena at test, whereas for the remaining animals, it was located at the bottom left corner. The same counterbalancing procedure was applied in *r*LL and control LL.

#### Visuospatial recognition

##### Spatial task 1.

The object-in-place recognition task was similar to the object recognition task, except that two different objects were placed at two corners of the arena (top left and bottom right) during the sample phase and the mouse was allowed to explore the objects freely for 10 min. After a 5 min or 24 h delay, the animal was tested for 1 min with new replicates of one preexposed object (Fig. 1*B*, middle row). One replicate was placed at the same spatial position as in the sample phase, whereas the other replicate was at a different spatial position (with respect to the visual cues on the walls of the arena). Animals normally spend more time exploring the replicate at the different location than the replicate at the same location, indicating encoding and retrieval of object–place associations (Dix and Aggleton, 1999). All other aspects of the object-in-place task, including the counterbalancing procedures, were identical to the object recognition task.

##### Spatial task 2.

In the object displacement task, two identical replicates of an object were placed at two corners of the arena (e.g., top left and bottom left) and the mouse was allowed to explore freely for 10 min. After the required delay, the animal was tested for 1 min with two new replicates of the same object. One replicate was at the same location as in the sample phase, whereas the other replicate was displaced to a new location that was not occupied by any object in the sample phase. Like the object-in-place task, it is anticipated that animals would spend more time exploring the replicate at the new location (Dix and Aggleton, 1999). The counterbalancing procedures were identical to the object recognition task.

#### Odor recognition

The odor recognition task was identical to the object recognition task except that the sample–test delay was always 5 min and shot glasses with essential oils rather than objects were presented in sample and test phases (Fig. 1*B*, bottom row).

#### Automated tracking of object and olfactory exploration

All sample and test phases were recorded via a web camera positioned above the center of the arena. Automated tracking of exploratory activity was conducted with ANY-maze software (version 4.5; Stoelting; RRID:SCR_014289). The tracking protocol used was similar to that in previous studies (Tam et al., 2014, 2015, 2016). For each video file, the 20 cm × 20 cm floor of the arena was outlined in ANY-maze, and 2 7 cm × 7 cm notional zones were placed at the corners of the arena where the objects were located. The position of the mouse's head within the arena was determined on a second-by-second basis and the amount of time its head was inside each of the two notional zones was recorded for every minute of the sample and test phases.

#### Data treatment

Memory performance on each recognition trial was expressed as a ratio score (*N* − *F*)/(*N* + *F*), where *N* and *F* represent the amount of time spent exploring novel versus familiar stimuli in the object and odor recognition tasks or objects at novel versus familiar locations in the object-in-place and object displacement tasks. The higher the ratio score is above zero (which indicates no stimulus discrimination), the better their recognition memory performance. Scores from the same task were averaged for each animal, resulting in one mean score for ZT/CT 6 and one mean score for ZT/CT 18 for each recognition task.

### Core body temperature assessment

**Figure 3.**
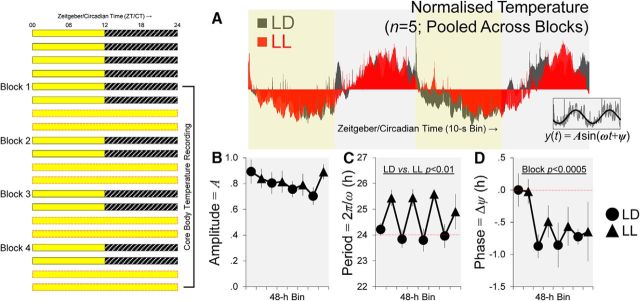
Core body temperature rhythms are maintained after four cycles of alternation between LD and LL in five mice. ***A***, Example of the normalized core body temperature rhythm pooled across blocks and mice. The mean ± SEM core body temperature prior to normalization was 36.29°C ± 0.14°C under LD, and it was 36.14°C ± 0.15°C under LL (pooled across blocks). Inset, Normalized time series from each 48 h period was fitted with a sinusoidal function, *A*sin(ω*t* + ϕ), to determine the amplitude (*A*), period length τ (2π/ω), and phase (ϕ) of each mouse's body temperature rhythm. ***B***–***D***, Mean values of the three parameters across 16 d of telemetry recording. There was no significant effect on amplitude values (***B***). The mean τ was longer during 48 h periods of LL than during LD (main effect of lighting condition, *p* < 0.01; ***C***) and there was a significant change in Δϕ across blocks (main effect of block, *p* < 0.0005; ***D***). These slight changes in circadian rhythms (i.e., period lengthening and phase delay of <1.5 h) cannot account for the complete reversal of odor recognition performance under *r*LL. Error bars indicate SEM in ***B***–***D***.

To investigate whether mice would maintain rhythmicity and nocturnality under the abnormal lighting condition, a naive cohort of mice were implanted with telemetry devices and, after recovery, their core body temperature was recorded continuously across multiple diurnal and circadian cycles for 16 d under *r*LL, as shown in Figure 3. We measured body temperature rather than wheel-running or rest-activity rhythms because constant light suppresses general activity, making it difficult to assess underlying circadian rhythms (Jud et al., 2005).

#### Surgery and telemetry equipment

During surgery, the mouse was anesthetized with isoflurane (IsoFlo; Abbott Laboratories,; 3% induction and 0.7–1.5% maintenance), and a telemetry transmitter (PhysioTel F20-EET; Data Sciences International) with a volume of 1.9 cm^3^ and a weight of 3.9 g was implanted into the peritoneum. Analgesics including buprenorphine (Vetergesic; Sogeval; 0.01 mg kg^−1^) and meloxicam (Metacam; Boehringer Ingelheim; 0.5 mg kg^−1^) were administered subcutaneously before surgery and 0.5 ml of saline was administered subcutaneously immediately after surgery to prevent dehydration; a second dose of meloxicam (0.5 mg kg^−1^) was given the next day. During recovery under LD and, for the duration of the experiment, telemetry-implanted animals were housed individually in transparent plastic cages (length × width × height = 48 cm × 26.5 cm × 21 cm) with *ad libitum* access to food and water. The cages were placed inside an LTC identical to that in the behavioral experiment except that the interior was shielded with aluminum foil to reduce interference. A telemetry receiver (PhysioTel RPC-1; Data Sciences International) was placed under each cage. When the implanted transmitters were switched on, signals received were transmitted to a hub (Data Exchange Matrix; Data Sciences International) and subsequently (via a local area network) to data acquisition software installed on a computer.

#### Data acquisition and processing

Implanted transmitters were switched on 6 weeks after surgery. Data acquisition began 5 min before ZT 0 (the start of the light phase under LD). As in the behavioral experiment, the lighting condition was alternated between 2 d of LD and 2 d of LL (Fig. 3). Over a period of 16 d, core body temperature was recorded continuously with a temporal resolution of 10 s using the Dataquest ART system (Data Sciences International). For each 2 d period under LD or LL, the time series in 10 s bins was smoothed using a 10 min moving average and converted to *Z* scores: (temperature in each bin − mean temperature)/standard deviation. The normalized time series was then fitted with a sinusoidal function, *A*sin(ω*t* + ϕ), using package nls2 in R (version 3.1.1; R Core Team, 2015), which enables us to determine the amplitude (*A*), period length τ (2π/ω), and phase (ϕ) of the mouse's core body temperature rhythm.

### *Per1*, *Per2*, and *Fos* gene expression

#### Brain tissue collection

A naive cohort of mice was housed either under LD or *r*LL conditions identical to that in the behavioral experiment (Fig. 1*A*). Brain tissue samples from the SCN, dorsal hippocampus, and olfactory bulb were collected at ZT 6 and 18 under LD and at CT 6 and 18 after four cycles of LD-LL alternation. At the required time of day, each mouse was removed from its home cage and killed by cervical dislocation under 100 lux of illumination, similar to that in the behavioral experiment. The eyes were removed immediately. The brain was removed from the skull, dipped in PBS to remove hair and blood, and then placed onto an ice-cold slicer matrix with 1 mm intervals (Zivic Instruments) with the ventral surface of the brain facing upward. Based on the Franklin and Paxinos (1997) mouse brain atlas, two skin graft blades (Swann-Morton) were placed at bregma −0.10 mm and −1.10 mm and SCN samples (located above the optic chiasm) were collected from this coronal section using a specimen punch with an internal diameter of 1 mm (Uni-Core; GE Healthcare). Two additional blades were placed at bregma −2.10 mm and −3.10 mm and hippocampal samples (including dorsal dentate gyrus, CA3, and CA1 subregions) were collected from these coronal sections. Finally, the entire olfactory bulb was collected from the brain. Samples were immediately frozen on dry ice and subsequently stored at −80°C before RNA extraction.

#### RNA extraction and real-time quantitative PCR (qPCR)

For each sample, total RNA was extracted and purified (RNeasy Mini Kit; QIAGEN) and RNA concentration was determined using the NanoDrop 1000 spectrophotometer (Thermo Scientific). Total RNA was converted into cDNA (qScript Synthesis Kit; Quanta Biosciences) and qPCR was performed in a thermocycler (StepOne Plus; Applied Biosystems) using SYBR Green I (QuantiFast SYBR Green PCR Kit; QIAGEN). The primer sequences 5′-3′ for *Per1* were AGTTCCTGACCAAGCCTCGTTAG (forward) and CCTGCCCTCTGCTTGTCATC (reverse); the sequences for *Per2* were GGGGTGAGATTCGTCATTGAACTTG (forward) and AGGACATTGGCACACTGGAAAGAG (reverse); and the sequences for *Fos* were ATCGGCAGAAGGGGAAAGTAG (forward) and GCAACGCAGACTTCTCATCTTCAAG (reverse). The sequences for the housekeeping gene β-actin were ACCAACTGGGACGATATGGAGAAGA (forward) and CGCACGATTTCCCTCTCAGC (reverse); the sequences for the housekeeping gene β2M were GCCTTCACCCCAGAGAAAGG (forward) and GCGGTTGGGATTTACATGTTG (reverse); and the sequences for the housekeeping gene *Gapdh* were TGCACCACCAACTGCTTAG (forward) and GATGCAGGGATGATGTTC (reverse). Melting curve analyses were performed to verify the specificity and identity of PCR products. Any sample with more than one peak in the first-derivative plot of the melting curve (which indicates primer-dimers or contaminating DNA) was excluded from subsequent analyses.

#### Quantification of gene expression

*Per1*, *Per2*, and *Fos* mRNA levels were quantified using the comparative threshold cycle (*C*_T_) method described by Schmittgen and Livak (2008). The 2^−CT^ values of *Per1*, *Per2*, and *Fos* in each sample were expressed as a ratio relative to the geometric mean of the 2^−CT^ values of the housekeeping genes in the same sample to obtain 2^−ΔCT^. The 2^−ΔCT^ values at ZT 6, CT 6, and CT 18 were then normalized to the mean 2^−ΔCT^ value of all samples from the same brain region at ZT 18 to obtain 2^−ΔΔCT^, which indicates the fold change in gene expression relative to midnight. Therefore, a 2^−ΔΔCT^ value >1 indicates that gene expression is elevated relative to ZT 18 under normal conditions, whereas a 2^−ΔΔCT^ value <1 indicates that gene expression is attenuated relative to ZT 18.

### Plasma corticosterone measurement

For a subgroup of mice from the gene expression experiment, we also collected blood samples and measured plasma corticosterone (CORT) levels at ZT 6 and 18 under LD and at CT 6 and 18 after four cycles of LD–LL alternation. The method of blood collection and CORT measurement are described in Pilorz et al. (2016). Briefly, trunk blood was obtained from the site of decapitation and collected into microcentrifuge tubes containing the anticoagulant ethylenediaminetetraacetic acid (EDTA; Sigma-Aldrich), with 5 μl of EDTA for every 250 μl of blood collected. Blood samples were kept on ice and centrifuged within 15 min after collection; the plasma obtained was stored at −20°C. Before CORT measurement, plasma samples were diluted 10-fold and CORT levels were determined with the AssayMax Corticosterone ELISA Kit (Assaypro) according to the manufacturer's instruction manual. Optical absorbance was read at 450 and 570 nm on a microplate reader (FLUOstar Omega; BMG Labtech) and the reading at the latter wavelength was subtracted from the former to correct for any optical imperfection. The standard curve relating optical absorbance to CORT concentration (in nanograms per milliliter) was derived by fitting a four-parameter logistic function.

### Statistical analyses

For recognition data collected under LD and *r*LL, two-way mixed ANOVAs were conducted on ratio scores and on total object exploration duration in each task, with lighting condition (LD or *r*LL) as a between-subjects factor and time of day (ZT/CT 6 or 18) as a within-subjects factor. For recognition data collected under control LL, a separate task (object, object-in-place, or odor) × time of day (CT 6 or 18) mixed ANOVA was conducted on ratio scores. Multiple one-sample *t* tests (two-tailed) were performed to compare mean recognition scores against the value of zero, which indicates no stimulus discrimination. For core body temperature data collected under *r*LL, block (days 1–4, 5–8, 9–12, or 13–16) × lighting condition (48 h LD or LL) within-subjects ANOVAs were conducted on amplitude (*A*), period length τ (2π/ω), and phase (ϕ), which were determined by nonlinear regression described above. For gene expression and plasma CORT data, lighting condition (LD or *r*LL) × time of day (ZT/CT 6 or 18) between-subjects ANOVAs were conducted on 2^−ΔΔCT^ and CORT concentration values, respectively. In all analyses, we adopted α = 0.05 for statistical significance (unless otherwise specified). Statistical analyses were conducted in IBM SPSS Statistics (version 22; RRID:SCR_002865).

## Results

### Object memory performance is optimal at midday

We first investigated whether performance in the object recognition task varied at ZT 6 versus 18 under LD and if such a time-of-day effect would persist at CT 6 versus 18 under *r*LL. Object memory performance was better at ZT 6 under LD and this time-of-day effect persisted under *r*LL (Fig. 1*B*, top row). A lighting condition [LD (*n* = 32) or *r*LL (*n* = 8)] × time of day (ZT/CT 6 or 18) ANOVA conducted on object recognition scores revealed that performance was better at ZT/CT 6 than at ZT/CT 18, as indicated by the significant main effect of time of day (*F*_(1,38)_ = 4.560, *p* < 0.05). However, the time-of-day effect on object performance did not differ between LD and *r*LL (main effect of lighting condition: *p* = 0.647; lighting condition × time of day interaction: *p* = 0.718). Under LD, the mean recognition scores at ZT 6 and 18 were both significantly different from the value of zero (both *p* < 0.0001), suggesting that mice were able to discriminate between novel and familiar objects at both times of day despite performance being better at ZT 6 than at ZT 18. Under *r*LL, the mean recognition score at CT 6 was significantly different from zero (*p* < 0.025), but the mean score at CT 18 was not different from chance (*p* = 0.196), further confirming that object recognition performance was optimal at subjective midday. Notably, the poorer performance at ZT/CT 18 was not a consequence of animals' reduced interest in object exploration per se. On the contrary, the total amount of time spent in object exploration in the sample phases was significantly higher at ZT/CT 18 (main effect of time of day: *F*_(1,38)_ = 7.985, *p* < 0.01; Table 2), although no day/night difference in overall exploratory activity was found in the test phases (*p* = 0.353; Table 3).

**Table 2.** Total exploration duration (s) in sample phases

Lighting condition	Time of day	Task							
		Object*^a^*		Object-in-place*^a^*		Object displacement*^a^*		Odor*^a^*	
		Mean	SEM	Mean	SEM	Mean	SEM	Mean	SEM
Condition 1: LD	ZT 06	91.235	7.364	78.236	6.767	140.750	8.844	56.035	5.907
	ZT 18	131.727	8.989	101.719	7.388	169.448	11.348	74.938	6.627
Condition 2: *r*LL	CT 06	105.192	14.433	74.016	5.813	116.284	8.614	62.031	5.939
	CT 18	124.230	9.935	79.204	5.073	124.478	8.989	76.099	6.092
Control LL	CT 06	123.087	13.557	95.660	9.303	—	—	56.405	8.120
	CT 18	132.898	11.587	101.521	7.614	—	—	79.565	10.156

*^a^*Significant main effects of time of day (*p* < 0.05).

**Table 3.** Total exploration duration (s) in test phases

Lighting condition	Time of day	Task							
		Object		Object-in-place		Object displacement		Odor	
		Mean	SEM	Mean	SEM	Mean	SEM	Mean	SEM
Condition 1: LD	ZT 06	30.984	2.051	21.756	3.077	31.556	2.554	18.219	2.202
	ZT 18	31.631	2.627	20.338	2.610	27.819	2.495	19.931	3.167
Condition 2: *r*LL	CT 06	16.988	2.271	18.513	1.639	24.908	1.578	14.217	1.827
	CT 18	10.825	2.191	15.163	1.953	27.413	1.782	19.583	2.340
Control LL	CT 06	24.644	3.364	29.913	4.954	—	—	6.775	1.502
	CT 18	31.431	2.812	33.525	5.013	—	—	9.738	3.086

### Visuospatial memory performance is optimal at midday

#### Spatial task 1: object-in-place task

Similar to the object recognition task, performance was better at ZT 6 in the object-in-place task under LD and this time-of-day effect persisted under *r*LL (Fig. 1*B*, middle row). A lighting condition [LD (*n* = 16)) or *r*LL (*n* = 16)] × time of day ANOVA conducted on spatial recognition scores revealed a main effect of time of day (*F*_(1,30)_ = 4.261, *p* < 0.05), but there was no main effect of lighting condition (*p* = 0.749) or lighting condition × time of day interaction (*p* = 0.335). At ZT 6 under LD and CT 6 under *r*LL, mean recognition scores were significantly different from zero (both *p* < 0.005), but mean scores at ZT 18 and CT 18 were not (*p* = 0.109 and 0.400, respectively), indicating that mice did not discriminate between different spatial positions at the latter time of day. Again, the level of object exploratory activity in the sample phases was higher at ZT/CT 18 (main effect of time of day: *F*_(1,30)_ = 6.323, *p* < 0.025; Table 2), but no day/night difference was found in the test phases (*p* = 0.244; Table 3).

#### Spatial task 2: object displacement task

In accordance with the findings from the object-in-place task, performance in the object displacement task was optimal at ZT/CT 6 (Table 4). As before, a lighting condition [LD (*n* = 16)) or *r*LL (*n* = 24)] × time of day ANOVA revealed a main effect of time of day (*F*_(1,38)_ = 4.395, *p* < 0.05), which did not differ between LD and *r*LL (lighting condition × time of day interaction: *p* = 0.866). Object exploratory activity in the sample phases was again higher at ZT/CT 18 (main effect of time of day: *F*_(1,38)_ = 5.820, *p* < 0.025; Table 2), but not in the test phases (*p* = 0.288; Table 3).

**Table 4.** Performance in the object displacement task

Lighting condition	Time of day	Ratio score*^a^*	
		Mean	SEM
Condition 1: LD	ZT 06	0.208	0.065
	ZT 18	0.061	0.109
Condition 2: *r*LL	CT 06	0.414	0.063
	CT 18	0.241	0.087

Recognition memory performance was expressed as a ratio score, (*N* − *F*)/(*N* + *F*), where *N* and *F* represent objects at novel versus familiar spatial positions. The higher the score is above zero, the better is the spatial recognition performance.

*^a^*Significant main effect of time of day (*p* < 0.025).

#### Short-term versus long-term memory performance

Evidence from invertebrate studies suggests that time-of-day effects on performance could vary depending on the delay interval between training and test (Fernandez et al., 2003; Lyons et al., 2005). To investigate whether this is the case for mice, we compared short-term (5 min sample–test delay) versus long-term memory performance (24 h sample–test delay) directly under LD. Similar time-of-day effects were found regardless of whether the test phase of each recognition trial was given 5 min or 24 h after the sample phase (Table 5). A three-way ANOVA was conducted, with sample–test delay [5 min (*n* = 16) or 24 h (*n* = 8)] as a between-subjects factor and task (object, object-in-place, or object displacement) and time of day (ZT 6 or 18) as within-subjects factors. There was a main effect of time of day as anticipated, with better performance at ZT 6 (*F*_(1,22)_ = 6.095, *p* < 0.025); however, it did not interact with delay (delay × time of day interaction: *p* = 0.814; delay × task × time of day interaction: *p* = 0.397). Long-term memory performance appeared to be poorer than short-term memory performance (Table 5), although the main effect of delay was not statistically significant (*p* = 0.101). Consistent with these results, the mean recognition score (pooled across tasks) at ZT 6 after a 24 h delay was significantly different from zero (*p* = 0.05), but the mean score at ZT 18 was not (*p* = 0.551), further confirming that day/night differences in object and visuospatial recognition performance persisted when the sample–test delay was increased to 24 h.

**Table 5.** Short-term versus long-term object and visuospatial performance

Sample–test delay	Time of day	Task					
		Object*^a^*		Object-in-place*^a^*		Object displacement*^a^*	
		Mean	SEM	Mean	SEM	Mean	SEM
5 min	ZT 06	0.451	0.076	0.317	0.093	0.208	0.065
	ZT 18	0.258	0.083	0.193	0.113	0.061	0.109
24 h	ZT 06	0.248	0.213	0.182	0.105	0.252	0.156
	ZT 18	0.229	0.104	−0.142	0.102	0.032	0.117

Recognition performance was expressed as a ratio score, (*N* − *F*)/(*N* + *F*), where *N* and *F* represent the amount of time spent exploring novel versus familiar objects or objects at novel versus familiar spatial positions. The higher the score is above zero, the better the recognition performance.

*^a^*Significant main effect of time of day (*p* < 0.05).

### Olfactory memory performance varies depending on lighting conditions

Strikingly, in contrast to object and visuospatial performance, the time-of-day effect in the odor recognition task varied between lighting conditions: Olfactory memory performance was optimal at ZT 6 under LD, but was optimal at CT 18 under *r*LL (Fig. 1*B*, bottom row). An ANOVA conducted on odor recognition scores, with lighting condition [LD (*n* = 8) or *r*LL (*n* = 24)] as a between-subjects factor and time of day as a within-subjects factor, showed that there was a significant interaction (*F*_(1,30)_ = 11.738, *p* < 0.0025). This interactive effect was due to the fact that, under LD, odor recognition performance was optimal at ZT 6 (simple main effect of time of day: *F*_(1,7)_ = 7.162, *p* < 0.05), similar to object and visuospatial performance. In contrast, under *r*LL, the time-of-day effect on performance was inverted. Olfactory performance was optimal at CT 18 (simple main effect of time of day: *F*_(1,23)_ = 10.604, *p* < 0.005). Furthermore, olfactory performance at CT 18 under *r*LL was significantly *enhanced* relative to ZT 18 under LD (simple main effect of lighting condition: *F*_(1,30)_ = 12.938, *p* < 0.005). Consistent with these results, the mean odor recognition score at ZT 6 under LD was significantly different from zero (*p* < 0.025), but mean at ZT 18 was not (*p* = 0.719); however, under *r*LL, the mean score at CT 18 was different from zero (*p* < 0.0001), but the mean at CT 6 was not (*p* = 0.223). Interestingly, this phase shift in olfactory performance was not accompanied by any shift in exploratory activity. Under both LD and *r*LL, odor exploratory activity in the sample phases was higher at ZT/CT 18, as indicated by the significant main effect of time of day (*F*_(1,30)_ = 9.585, *p* < 0.005; Table 2), which did not interact with lighting condition (*p* = 0.653). No day/night difference in exploratory activity was found in the test phases (*p* = 0.171; Table 3).

### Forty-eight-hour LL does not alter the circadian phase of olfactory memory performance

To investigate whether the phase shift in olfactory performance was due to repeated alternation between LD and LL or prolonged exposure to light, a cohort of naive mice were given only a single 48 h period of light as a control condition (Fig. 2*A*). In contrast to the results observed under *r*LL, in the control condition, performance in the object task (*n* = 16), the object-in-place task (*n* = 8), and the odor task (*n* = 16) was consistently better at CT 6 (main effect of time of day: *F*_(1,37)_ = 4.935, *p* < 0.05), similar to that under LD. Crucially, this day/night difference in performance did not vary across the three tasks (task × time of day interaction: *p* = 0.370; Fig. 2*B*). This indicates that a one-off, 48 h exposure to light was not sufficient to induce a complete reversal of olfactory performance at CT 6 and 18. However, there was a main effect of task (*F*_(2,37)_ = 3.471, *p* < 0.05) due to the fact that performance in the odor task was generally poorer than performance in the object task in these animals (Bonferroni *post hoc* comparison, *p* < 0.05; Fig. 2*B*).

### Circadian rhythms are maintained under *r*LL

To confirm that mice maintained nocturnality as well as rhythmicity under *r*LL, core body temperature was recorded continuously across multiple diurnal and circadian cycles in five telemetry-implanted mice. An example of the core body temperature rhythm is shown in Figure 3*A*; mean amplitudes (*A*), period lengths τ (2π/ω), and phase changes (Δϕ) across days are displayed in Figure 3, *B–D*, respectively. Visual inspection of Figure 3*A* suggests that mice maintained rhythmicity and nocturnality under *r*LL, with a trough and a peak during the (subjective) day and night, respectively. Furthermore, the internal period (the duration from one peak to the next) was lengthened under LL (Aschoff, 1960). These observations were confirmed by ANOVAs, which showed that mean amplitudes did not vary significantly across days (Fig. 3*B*). However, as predicted by Aschoff's rule, the mean τ was longer under LL (main effect of lighting condition: *F*_(1,4)_ = 25.039, *p* < 0.01; Fig. 3*C*); in addition, it was significantly longer than 24 h (*p* < 0.005). This increase in τ under LL led to a significant phase delay across days (main effect of block: *F*_(3,12)_ = 14.466, *p* < 0.0005; block × lighting condition interaction: *F*_(3,12)_ = 97.294, *p* < 0.0005; Fig. 3*D*). Nevertheless, these slight changes in circadian rhythms (i.e., period lengthening and phase shifting of <1.5 h) do not explain the complete reversal of the time-of-day effect on olfactory performance under *r*LL.

### Gene expression in the SCN, dorsal hippocampus, and olfactory bulb is differentially affected by *r*LL

**Figure 4.**
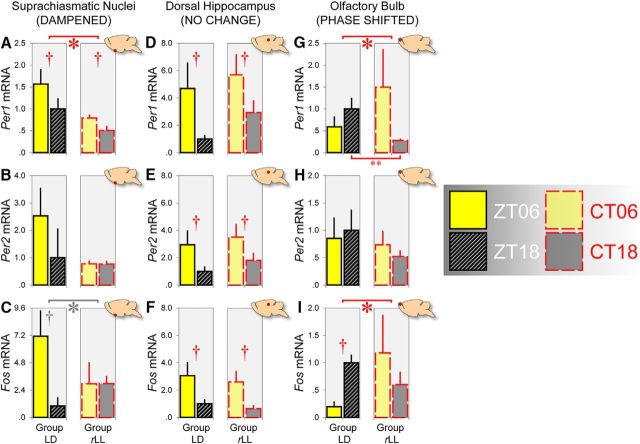
*r*LL produces differential effects on the canonical clock genes *Per1* and *Per2* and the immediate-early gene *Fos* mRNA expression in the SCN (DAMPENED), dorsal hippocampus (NO CHANGE), and olfactory bulb (PHASE SHIFTED). ***A***–***C***, Gene expression in the SCN was dampened in mice housed under *r*LL after 4 cycles of LD–LL alternation (*n* = 6 at CT 6 and *n* = 6 at CT 18 for each gene) relative to animals housed under LD (*n* = 6 at ZT 6 and *n* = 6 at ZT 18 for each gene). Red asterisk and daggers in ***A*** indicate the main effect of lighting condition (*p* < 0.05) and main effect of time of day (*p* < 0.005), respectively, whereas gray asterisk and dagger in ***C*** indicate the marginally significant lighting condition × time of day interaction and marginal main effect of time of day (both *p* = 0.057). ***D***–***F***, Mean *Per1*, *Per2*, and *Fos* mRNA levels in the hippocampus were unaffected by *r*LL. Gene expression was consistently higher at ZT 6 than at ZT 18 for mice housed under LD (*Per1* and *Per2*: *n* = 15 at ZT 6 and *n* = 14 at ZT 18; *Fos*: *n* = 6 at ZT 6 and *n* = 6 at ZT 18) and higher at CT 6 than at CT 18 for mice housed under *r*LL (*Per1*: *n* = 10 at CT 6 and *n* = 12 at CT 18; *Per2*: *n* = 10 at CT 6 and *n* = 14 at CT 18; *Fos*: *n* = 6 at CT 6 and *n* = 5 at CT 18). Daggers in ***D***–***F*** indicate main effects of time of day (all *p* < 0.025). ***G***–***I***, Gene expression in the olfactory bulb was phased shifted in mice housed under *r*LL (*Per1*: *n* = 10 at CT 6 and *n* = 14 at CT 18; *Per2*: *n* = 12 at CT 6 and *n* = 14 at CT 18; *Fos*: *n* = 4 at CT 6 and *n* = 6 at CT 18) relative to animals housed under LD (*Per1*: *n* = 13 at ZT 6 and *n* = 15 at ZT 18; *Per2*: *n* = 16 at ZT 6 and *n* = 15 at ZT 18; *Fos*: *n* = 6 at ZT 6 and *n* = 6 at ZT 18). Asterisks in ***G*** and ***I*** indicate lighting condition × time of day interactions (*p* ≤ 0.05); double asterisk in ***G*** indicates the simple main effect of lighting condition at ZT/CT 18 (*p* < 0.01); dagger in ***I*** indicates the simple main effect of time of day under LD (*p* < 0.005). Error bars indicate SEM in all panels.

To understand how the abnormal lighting condition would affect molecular rhythms in different brain regions, we measured mRNA expression of two canonical clock genes, *Per1* and *Per2*, which regulate behavioral rhythms (Bae et al., 2001; Reppert and Weaver, 2002). In addition, we looked at mRNA expression of the immediate-early gene *Fos*, a marker of neuronal activity. Mean *Per1*, *Per2*, and *Fos* mRNA levels in different regions under LD versus *r*LL (after four cycles of LD–LL alternation) are displayed in Figure 4.

#### SCN

We found that *Per1* mRNA levels in the SCN were higher at ZT 6 than at ZT 18 under LD and higher at CT 6 than at CT 18 under *r*LL (main effect of time of day: *F*_(1,20)_ = 5.023, *p* < 0.05; Fig. 4*A*). Moreover, *Per1* mRNA expression was generally lower under *r*LL relative to LD (main effect of lighting condition: *F*_(1,20)_ = 10.997, *p* < 0.005), consistent with previous findings that prolonged periods of LL dampen circadian rhythms in the SCN (Beaulé et al., 2003; Sudo et al., 2003; Granados-Fuentes et al., 2004; Coomans et al., 2013). However, the lighting condition × time of day interaction was not significant (*p* = 0.469), suggesting that *r*LL dampened, but did not completely abolish, the rhythmic expression of *Per1* in the SCN. Similarly, *Fos* mRNA expression was higher at ZT 6 than at ZT 18 under LD and this day/night difference was attenuated under *r*LL (Fig. 4*C*), although these effects were only marginally significant (main effect of time of day: *F*_(1,20)_ = 4.065, *p* = 0.057; lighting condition × time of day interaction: *F*_(1,20)_ = 4.088, *p* = 0.057).

#### Dorsal hippocampus

In contrast to the weakening of molecular rhythms in the SCN, *r*LL did not dampen *Per1*, *Per2*, or *Fos* mRNA levels in the dorsal hippocampus, a region that (together with adjacent and interconnected cortical areas) contributes to object and visuospatial recognition memory processes (Aggleton et al., 2012; Cohen et al., 2013). Hippocampal gene expression was elevated at ZT 6 and CT 6, as indicated by significant main effects of time of day (*Per1*: *F*_(1,47)_ = 6.004, *p* < 0.025; *Per2*: *F*_(1,49)_ = 6.115, *p* < 0.025; *Fos*: *F*_(1,19)_ = 9.356, *p* < 0.01; Fig. 4*D–F*, respectively). However, there was no main effect of lighting condition (*Per1*, *p* = 0.269; *Per2*, *p* = 0.358; *Fos*, *p* = 0.545) or lighting condition × time of day interaction (*Per1*, *p* = 0.724; *Per2*, *p* = 0.869; *Fos*, *p* = 0.940), indicating that hippocampal gene expression was unaffected under *r*LL. These molecular data mirror the behavioral observations that object and visuospatial recognition performance were unaffected under *r*LL.

#### Olfactory bulb

Strikingly, *Per1* mRNA expression patterns in the olfactory bulb were phase shifted under *r*LL, as indicated by a significant lighting condition × time of day interaction (*F*_(1,48)_ = 4.733, *p* < 0.05). This is due to the fact that there was a significant decrease in *Per1* mRNA levels at CT 18 under *r*LL relative to ZT 18 under LD (simple main effect of lighting condition: *F*_(1,27)_ = 8.349, *p* < 0.01; Fig. 4*G*). A similar phase shift was found for *Fos* mRNA expression under *r*LL (lighting condition × time of day interaction: *F*_(1,18)_ = 4.419, *p* = 0.05; Fig. 4*I*). These changes in *Per1* and *Fos* mRNA expression correspond to the behavioral observation that odor recognition performance was phase shifted under *r*LL.

### Plasma corticosterone levels are reduced following *r*LL

Prolonged exposure to LL is known to reduce the level of arousal in mice, typically assessed by measuring plasma CORT (Fonken et al., 2010; Coomans et al., 2013). To investigate whether *r*LL would reduce the level of CORT as in previous studies, in a subgroup of mice from the gene expression experiment, we collected blood samples and measured plasma CORT levels at ZT 6 and 18 under LD and at CT 6 and 18 after 4 cycles of LD–LL alternation. Consistent with previous studies, the mean CORT concentration under *r*LL (4.047 ± 0.637 ng/ml) was significantly lower than the mean under LD (7.274 ± 1.551 ng/ml) (main effect of lighting condition: *F*_(1,16)_ = 7.910, *p* < 0.025). CORT concentrations were slightly higher at (subjective) night, as expected in nocturnal rodents (ZT 6 vs ZT 18: 6.442 ± 2.628 ng/ml and 8.105 ± 2.628 ng/ml, respectively; CT 6 vs CT 18: 3.745 ± 0.898 ng/ml and 4.350 ± 0.970 ng/ml, respectively). However, these day/night differences were not statistically significant (main effect of time of day: *p* = 0.127; lighting condition × time of day interaction: *p* = 0.590).

## Discussion

Here, we demonstrate that there are diurnal rhythms in recognition memory for objects, places, and odors, with performance consistently better at ZT 6 regardless of the delay interval between sample and test phases. Strikingly, these rhythms in performance become desynchronized under *r*LL. Object and visuospatial performance remains better at CT 6, but olfactory performance becomes better at CT 18. This desynchrony in behavioral performance is mirrored by differential changes in *Per1*, *Per2*, and *Fos* mRNA expression in the SCN, dorsal hippocampus, and olfactory bulb. Temporal desynchrony among these regions does not result in arrhythmicity because core body temperature and exploratory activity rhythms persist under *r*LL. To our knowledge, this is the first demonstration that abnormal lighting conditions can give rise to internal desynchrony at the level of circadian oscillators in different brain regions and at the level of behavioral performance in object, visuospatial, and odor recognition tasks without inducing arrhythmicity or disrupting memory performance (Ruby et al., 2008, 2013; Fernandez et al., 2014).

Our finding that memory performance is generally better at ZT/CT 6 is consistent with previous studies, which reported optimal performance in the light phase in aversively motivated and appetitively motivated tasks (Chaudhury and Colwell, 2002; Rawashdeh et al., 2014; but see Valentinuzzi et al., 2001; Gritton et al., 2009, 2012, for different results). So why is recognition performance generally better at ZT/CT 6? A potential factor that could have contributed to our behavioral results is arousal—either the absolute level or differential change in arousal—induced by handling or removal from the home cage, resulting in differential levels of performance at ZT/CT 6 and 18. Nevertheless, because the form of the arousal–performance relationship often follows an inverted-U function (Yerkes and Dodson, 1908; Broadhurst, 1957; Diamond et al., 2007), it is unclear whether the day/night difference in performance was caused by a facilitative effect at ZT/CT 6 due to a moderate change in arousal from a previously inactive state, a disruptive effect at ZT/CT 18 due to a further elevation in the absolute level of arousal from a previously mildly active state, or both. This issue can only be resolved in future studies in which physiological measures of arousal (such as heart rate) are assessed before and during behavioral testing at ZT/CT 6 and 18.

Another factor that could have contributed to our behavioral results is the amount of sleep, which is regulated by circadian cycles as well as preceding sleep–wake history (Borbély et al., 2016). Importantly, sleep–wake history between sample and test phases is known to be a crucial determinant of performance, especially when the sample–test delay is relatively long (1.5–24 h). For example, forced wakefulness manipulations immediately after object preexposure, such as handling (Palchykova et al., 2006; Inostroza et al., 2013) and stimulation of arousal-related pathways (Rolls et al., 2011), are known to disrupt long-term recognition performance, whereas sleep after object preexposure facilitates visuospatial performance (Binder et al., 2012). These findings are consistent with the general idea that posttraining sleep may be important for reactivation of memory traces (Buzsáki, 1998; Ribeiro and Nicolelis, 2004; Rasch and Born, 2013). Nevertheless, although the role of sleep in memory consolidation is potentially relevant to our results on trials with a 24 h sample–test delay, it is unlikely to be relevant to our results on trials with a 5 min delay because sleep-dependent memory consolidation would not be necessary for successful short-term stimulus recognition (Squire et al., 2015).

Alternatively, the day/night difference in short-term memory performance may be partly related to sleep-driven synaptic homeostasis (Cirelli and Tononi, 2000; Tononi and Cirelli, 2003), leading to differences in synaptic weights in the relevant brain circuitries involved in recognition memory processes. One possible factor is the diurnal rhythm in GluA1-containing AMPA receptors in the postsynaptic density, which reflects an animal's preceding sleep–wake history (Vyazovskiy et al., 2008). Notably, GluA1-mediated synaptic plasticity may, at least in part, underlie the short-term recognition memory processes that we have investigated in the present study because GluA1 deletion selectively disrupts short-term object, spatial, and olfactory memory performance while sparing long-term memory (Sanderson et al., 2011; Sanderson and Bannerman, 2012; Ang, 2016). However, the exact relationship between diurnal variation in synaptic GluA1 levels driven by preceding sleep–wake history and subsequent memory performance is not known. Future studies in which sleep electroencephalogram and synaptic GluA1 levels are assessed can help to confirm the effects of preceding sleep–wake history on GluA1-dependent recognition memory performance. Nevertheless, synaptic homeostasis per se is not sufficient to account for the fact that *r*LL desynchronizes daily rhythms in olfactory versus object and visuospatial performance.

The decoupling of behavioral performance suggests that synaptic changes required for these recognition processes are regulated by distinct circadian oscillators (Herzog, 2007; Frank, 2016). More specifically, under LD, *Per1*, *Per2*, and *Fos* are all rhythmically expressed in the dorsal hippocampus, with higher expression at ZT 6, highly similar to the phasing of gene expression in the SCN as in previous studies (Gilhooley et al., 2011; Reale et al., 2013; Loh et al., 2015; Ma et al., 2016; but see Wang et al., 2009; Jilg et al., 2010; Harbour et al., 2014, for different results). These genes are also rhythmically expressed in the olfactory bulb, but are in antiphase to the gene expression profile in the SCN (Abe et al., 2002; Abraham et al., 2005; Granados-Fuentes et al., 2006). Crucially, under *r*LL, *Per1* and *Fos* rhythms in the SCN are dampened, although not completely abolished, consistent with previous findings that SCN rhythms are compromised by prolonged periods of LL (Beaulé et al., 2003; Sudo et al., 2003; Granados-Fuentes et al., 2004; Coomans et al., 2013). In contrast, under *r*LL, hippocampal gene expression is unaffected, whereas *Per1* and *Fos* expression in the olfactory bulb is phase shifted.

These data are consistent with previous findings that the olfactory bulb expresses autonomous and entrainable circadian rhythms, which persist independently of the SCN (Abe et al., 2002; Granados-Fuentes et al., 2004; Abraham et al., 2005; Granados-Fuentes et al., 2006; Guilding and Piggins, 2007). Circadian variation in olfactory discrimination performance persists in SCN-lesioned mice that exhibit arrhythmicity in wheel-running activity; however, the circadian phase of optimal olfactory discrimination is shifted relative to control animals (Granados-Fuentes et al., 2011). Our data complement and extend these observations by showing that abnormal lighting conditions can suppress the influence of the SCN, giving rise to internal desynchrony at the level of circadian oscillators in different brain regions and at the level of behavioral performance in different sensory domains.

We cannot rule out the possibility that changes in the circadian phase of olfactory processing under *r*LL could be the result of altered feeding behavior because prolonged LL can induce a phase shift in feeding behavior (Fonken et al., 2010), potentially leading to phase shifts in *Per1* and *Per2* rhythms in food-entrainable oscillators among different extra-SCN regions (Wakamatsu et al., 2001) and changes in behavioral performance (Loh et al., 2015). However, in these studies, altered feeding was also accompanied by arrhythmicity (Fonken et al., 2010; Loh et al., 2015) and phase shifts in hippocampal *Per1* and *Per2* rhythms (Wakamatsu et al., 2001). Notably, neither of these effects was observed in our study, suggesting that it is unlikely that feeding behavior was altered by *r*LL.

**Figure 5.**
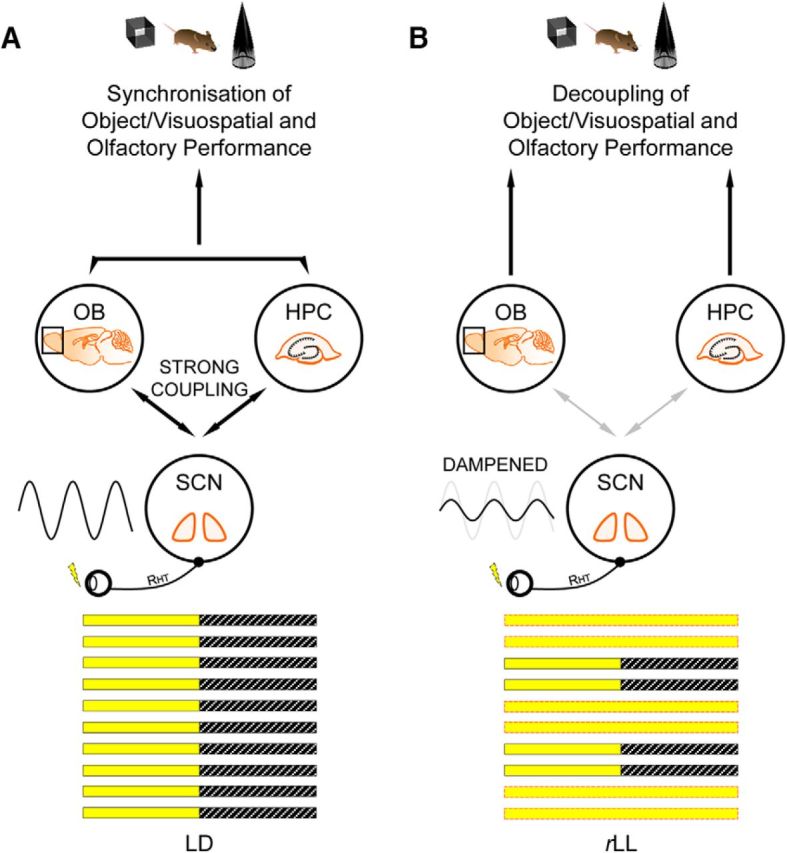
Model describing the effect of *r*LL on the SCN and its knock-on effect on peripheral oscillators. ***A***, Under LD, circadian oscillators in the olfactory bulb and hippocampus are tightly coupled to the central SCN pacemaker, allowing extra-SCN oscillators to maintain a constant phase relationship with the SCN, as well as with one another. ***B***, Under *r*LL, the central SCN pacemaker is dampened due to abnormal light inputs and this weakens the coupling between the SCN and extra-SCN oscillators. Without a strong coupling with the SCN, the circadian oscillator in the olfactory bulb becomes autonomous, resulting in a shift in the circadian phase of odor recognition performance. This model is based on multiple sources, including Herzog and Tosini (2001), Guilding and Piggins (2007), Dibner et al. (2010), Kyriacou and Hastings (2010), and Granados-Fuentes et al. (2011). Schematics of the mouse brain are adapted from the PPT Drawing Toolkit–Neuroscience (illustrations 7111409 and 7111412; Motifolio). HPC, Hippocampus; OB, olfactory bulb; RHT, retinohypothalamic tract.

Our results can be understood in terms of the hierarchical organization of the circadian system, with the SCN playing a key role in maintaining entrainment of peripheral oscillators (Dibner et al., 2010). Under LD, peripheral clocks in the olfactory bulb and hippocampus are coupled to the SCN or its downstream signals such as circulating glucocorticoids (which may synchronize peripheral oscillators via mineralocorticoid and glucocorticoid receptors; Balsalobre et al., 2000). This results in optimal object, visuospatial, and olfactory recognition performance at ZT 6 (Fig. 5*A*). However, under *r*LL, molecular rhythms in the SCN and its downstream signals (e.g., CORT) are dampened, weakening the coupling between the SCN and extra-SCN regions. The olfactory bulb becomes autonomous from the SCN, leading to a shift in the circadian phase of olfactory processing (Fig. 5*B*). In contrast, the oscillator in the hippocampus, and presumably oscillators in adjacent and interconnected cortical areas that also contribute to object and visuospatial processing, may depend upon signals from the SCN to drive performance. It should be noted that molecular oscillations in the hippocampus may also be autonomous under certain conditions, although previous studies were inconclusive (Abe et al., 2002; Wang et al., 2009; Ma et al., 2016).

What are the adaptive advantages of multiple dissociable oscillators? In nature, where resources and predators are encountered at varying times of day, anticipating the temporal regularities of these different events is essential to survival. Multiple clocks that adopt different phase relationships with one another may enable processes occurring in different regions to be optimized to specific phases of the 24 h cycle (Antle and Silver, 2009). Moreover, such a dispersed network of circadian oscillators may provide greater flexibility when faced with conflicting environmental signals.
